# Achieving Response With Bolus Dosing of Valproate in Electroconvulsive Therapy (ECT)-Induced Mania: A Case Report

**DOI:** 10.7759/cureus.77395

**Published:** 2025-01-13

**Authors:** Venkatesh Sreeram, Nana Bonsu

**Affiliations:** 1 Psychiatry, Augusta University Medical College of Georgia, Augusta, USA

**Keywords:** antipsychotics, ect, ect-induced mania, electroconvulsive therapy (ect), lithium, mania, valproate

## Abstract

Electroconvulsive therapy (ECT) is an effective treatment for various mental illnesses. While it is an excellent modality in treating depression and severe mania, it can also induce mania in certain specific settings. There is so far scarce literature regarding the management of ECT-induced mania with antipsychotics and mood stabilizers, specifically lithium, utilized in different studies. Moreover, there is limited evidence for using valproate for the symptoms. We present a case vignette about managing mania symptoms after ECT by administering a bolus dose of valproate at each session, resulting in progressively improved outcomes.

## Introduction

Electroconvulsive therapy (ECT) is an effective treatment modality in psychiatry. ECT can help with depression, bipolar disorder, catatonia, and associated psychotic features. However, some studies in the literature suggest that ECT can induce mania, the prevalence of which can be as high as 25% [1]. These studies recommend the discontinuation of ECT if an individual presents with mania, although no specific guidelines exist on the management [1-2]. We present a complex case where ECT as maintenance therapy may have induced mania, despite the patient being on mood stabilizers and managed with valproate.

## Case presentation

A 53-year-old male with a history of schizoaffective disorder who was on ECT maintenance along with complex psychopharmacology was living in a structured environment like a personal care home. He presented to a crisis center due to increasing symptoms, including alarming signs such as rapid speech, grandiose delusions, inappropriate sexual behavior toward females, and lack of sleep. He eventually got hospitalized due to active symptoms, which were beyond the capacity of the personal care home to manage. The issues in his mental status were evident with slurred and hyperverbal speech, intrusiveness, disruptive behavior, grandiose thoughts, and increased energy. Physical examination and laboratory results were unremarkable other than tachycardia.

The patient's past psychiatric history included a chronic history of psychosis and approximately 24 psychiatric hospitalizations within the same state psychiatric facility. He tested negative for substance use. The treatment team, well aware of his symptoms and outcomes from past hospitalizations, provided comprehensive care. Along with ECT, his medication regimen included clozapine for his psychotic symptoms, valproate for mood stabilization, lorazepam for excited catatonia, gabapentin for anxiety, and trazodone for insomnia. Clozapine and valproate serum levels were in therapeutic ranges.

He was getting weekly ECT maintenance sessions as an outpatient; tapering ECT in the past had not been successful, as he had developed breakthrough symptoms of depression and psychosis. Hence, ECT was continued at the same pace during the hospitalization. The treatment team noted him having breakthrough symptoms of mania after ECT treatment, which lasted for one to two days post-ECT, including lack of sleep, being hyperverbal, intrusive, irritable, agitated, being sexually inappropriate with females, and using profanities. The providers that performed ECT sessions reported him having agitation and aggression post-treatment, almost as if he had not undergone an ECT session.

Notably, this had become a pattern post-ECT, and his mood, sleep, and behavior returned to normal following one to two days of hypomanic symptoms. While there was a suspicion of treatment not being sufficient or that he was becoming resistant to ECT, the pattern of manic symptoms after ECT sessions indicated that the treatment may have been inducing the symptoms. On gathering the collateral information, it was found that a similar pattern had happened over the last six months, and the patient no longer found ECT helpful. However, the patient and the family were educated about continuing the ECT, given that treatment without ECT sessions was unsuccessful.

The frequency of ECT sessions increased from weekly to 10 days as per individual request, and his behavior improved for a week, followed by two days of manic symptoms post-ECT. However, the patient showed poor tolerance to ECT given every 10 days, resulting in breakthrough symptoms. Hence the frequency was reverted to weekly ECT sessions. According to the providers performing the ECT, his seizure duration was good.

The team then felt that the valproate dose was insufficient, the dose of which was being withheld before ECT due to the interaction with the seizure duration. Hence, we attempted to give a bolus dose of valproate following the ECT session to prevent mania symptoms. We followed this pattern after having a discussion with the ECT-providing team for three months, and this modality seemed adequate, as the symptoms were hugely controllable, and his behavior improved. Eventually, the bolus dosing was discontinued; his liver function was intact, and valproate levels were maintained at 75-95 mg/l during bolus dosing. The team discharged him to the personal care home, although long-term monitoring of the effectiveness to assess the need to reconsider bolus dosing may be warranted.

## Discussion

ECT has been considered an effective modality to treat severe acute mania [3-4]. Our patient, however, developed manic/hypomanic symptoms following ECT. His previous hospitalizations were in the setting of discontinuing maintenance ECT, which was not the case with the current hospitalization. While there is a possibility of him having a natural progression of bipolar disorder or going through a rapid-cycling disorder, we considered it to be low due to the history of him strictly adhering to the medication regimen and lab testing. Also, the pattern of symptoms appearing between ECT sessions convinced the entire team that ECT would have caused the symptoms.

There is evidence to show that using lithium during such instances is helpful [2,5-6]. However, there are limited studies about the use of valproate along with ECT to control mania symptoms [7,8]. Our patient had already tried lithium in the past and could not tolerate it due to renal complications; hence, he was maintained on valproate. There is data in the literature about antipsychotic medications used during ECT, which would also provide additive benefits in controlling the symptoms [9]. Given that the patient was already being maintained on clozapine at a reasonable therapeutic level, the team was apprehensive about adding a new antipsychotic medication to avoid further interactions.

Based on the findings in the vignette, ECT seemed to cause the mania symptoms, although it cannot be definitively concluded as the only cause. We could not use any rating scales to document the progression of symptoms in this case. As per Thomas et al., ECT can be used as a mood-stabilizing treatment when mania is induced, which also seemed to be the case in our vignette [10]. The alternative reasons for mania symptoms in our patient would be holding off valproate and lorazepam doses a day before ECT. Despite the valproate levels being therapeutic at 78 mg/L at the admission, holding a dose or two could probably bring about the symptoms; however, no studies were available to substantiate it. Trazodone could be another factor that can induce mania symptoms. However, he had been maintained on a 100 mg dose of trazodone for years before without any similar symptoms.

There was limited or no substantial evidence available regarding administering a bolus dose of mood stabilizer following ECT to benefit the individuals in preventing mania. However, one case study has described the use of valproate following ECT-induced mania, which led the individual to become depressed, and lithium, in contrast, seemed to show benefit [3,10]. While there was a risk of toxicity associated with giving a bolus dose in such cases, no such reports were available, and the levels were maintained at therapeutic range despite the higher dose after each session.

Several case studies are available in the literature on ECT-induced mania, where depression was the primary diagnosis [11-12]; however, there are scarce studies on bipolar-type individuals presenting with mania symptoms after ECT. Although the relationship between ECT and mania has been touched on in the literature, no direct causal relationship has been established [13-14].

## Conclusions

Although ECT is an effective modality in various mental illnesses, its use has been linked to several challenges, including the requirement of general anesthesia and informed consent and the stigma associated with the procedure. Although no direct causal relationship between ECT and developing mania exists, the literature suggests that the risk is higher with early age of onset and long duration of the illness. There are no strict guidelines suggesting that ECT-induced manic symptoms would require treatment with mood stabilizers or antipsychotics, although both seem to have good benefits as per the studies. While these agents are helpful in various settings, the question arises as to whether the treatment is required only during acute settings or if continued maintenance treatment is needed. Limited studies have shown evidence for the long-term use of these agents in such situations.

While ECT seemed to be the cause of the symptoms in our vignette, it could be a natural progression of bipolar disorder, or the patient may have rapid-cycling bipolar disorder, which is a limitation of this report. Other limitations include not using any rating scales for assessing improvement and relying on follow-up evaluations and therapeutic levels of the medication. No studies have yet proposed bolus doses of medications to control ECT-induced mania, and discontinuation of ECT did not show the resolution of symptoms. Hence, more studies that involve bolus dosing of the medications and monitoring their therapeutic levels in these patients are required if other options do not work.
